# Subtotal cholecystectomy: An operative option for Salmonellae typhi gangrenous cholecystitis

**DOI:** 10.1002/ccr3.3130

**Published:** 2020-07-15

**Authors:** Vivian Valin Akello, Denis Bitamazire, Joel Wandabwa, Robinson Ssebuufu

**Affiliations:** ^1^ Department of Surgery Kampala International University Bushenyi Uganda; ^2^ Department of Surgery Hoima Regional Referral Hospital Hoima Uganda

**Keywords:** gangrenous cholecystitis, paediatric surgery, subtotal cholecystectomy, typhoid fever

## Abstract

Typhoid gangrenous cholecystitis is uncommon and can be managed by subtotal cholecystectomy with nonclosure of the cystic duct if that is required for patient safety.

## Graphical abstract

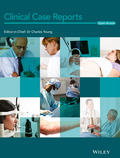

## 1 INTRODUCTION

The occurrence of gangrenous cholecystitis as a complication of typhoid fever is rare. This is a case study of a 7‐year‐old girl with typhoid fever diagnosed with gangrenous cholecystitis and gallbladder empyema and successfully managed by subtotal cholecystectomy and nonclosure of the cystic duct.

Typhoid fever is endemic in the tropics; however, the prevalence is difficult to estimate since many febrile illnesses like malaria present similarly.^1^ Humans are the only natural host and reservoir of *Salmonella Typhi* (S Typhi) which is transmitted by the fecal‐oral route.^1^ In endemic areas like Uganda, it is predominately common in children aged 5‐19 years, possibly due to their immature immune system.^2^ Gangrenous cholecystitis is rare sequelae of acalculous cholecystitis occurring in patients with typhoid fever. We present a case of a complicated typhoid fever in a child. Approval for publication was sought from the ethics committee, and parental consent was requested to publish the case details.

## 2 CASE PRESENTATION

N.T a 7‐year‐old girl presented to Hoima Regional Referral Hospital (HRRH) in northwestern Uganda as a referral from a district hospital following five days of hospitalization. She had been unwell for 13 days prior to admission with high‐grade fevers and abdominal pain. She received symptomatic treatment for a probable diagnosis of malaria and septicemia which was treated with coartem, ampicillin, and gentamicin prior to her admission at HRRH. She had recently developed abdominal distension in the last two days which prompted the referral. She had no history of yellowing of eyes. She reported episodes of loose motions and no constipation. The attendant also reported poor appetite and loss of weight.

On examination, she was febrile (38‐degree Centigrade), mildly wasted, had no jaundice, and was not dehydrated. Examination of the abdomen revealed moderate distension with generalized and rebound tenderness. The bowel sounds were reduced. A diagnosis of generalized peritonitis was made, and an exploratory laparotomy was scheduled. The chest X‐ray done was normal, abdominal ultrasound showed free intra‐abdominal fluid, complete blood count showed leukocytosis (14 900 cells/µL) and anemia (8.0 g/dL), and Widal test was positive. Preoperatively, the patient was resuscitated with normal saline and dextrose, started on metronidazole and ceftriaxone and given rectal paracetamol.

At exploratory laparotomy, we found edematous, thickened bowel that was matted with purulent ascites of about 200 mL and no gut perforation. The omentum was adherent to the surface of the liver. A gangrenous gallbladder with a small gallbladder empyema of about 30 mL of purulent fluid was identified. We experienced difficulty in dissecting Calot's triangle due to adhesions and necrotic gallbladder wall, and a decision was made to do a subtotal cholecystectomy with nonclosure of the cystic duct. We left a subhepatic and pelvic drain in situ. On the second postoperative day, the child developed a bile leak (about 100 mL per day) that subsequently reduced. The leakage was managed conservatively with antibiotics (ceftriaxone and metronidazole) and early resumption of oral feeds on the second postoperative day. On the fourth postoperative day, the pelvic drain was removed and the subhepatic drain removed 8 days later. Thereafter, the child was discharged.

## 3 DISCUSSION

Gangrenous gallbladder following S. Typhi or in association with S Typhi is a rare occurrence in children. Ileal perforation is the commonest complication of typhoid managed surgically. Cholecystitis associated with typhoid has an incidence of 2.8%‐12.5% though this figure may rise with routine use of ultrasound scan in typhoid patients.^3^

*Salmonella Typhi* cholecystitis in children occurs between the ages of 5 and 19 years, often in the second week of illness.^4^ Gallbladder disease was not suspected in this patient who was operated with a generalized peritonitis in mind. This is similar to a study done in Nigeria where three children with typhoid gangrenous cholecystitis were thought to have typhoid enteritis with peritonitis before exploratory laparotomy.^5^ Sonographic findings of thickened gallbladder wall with thickened bowel wall especially ileum and cecum and multiple mesenteric nodes are diagnostic of typhoid fever in endemic areas.^6^ Ultrasound scan should be encouraged especially in typhoid endemic areas for early diagnosis and where necessary use of less invasive management options. Though we did not get culture and sensitivity results, the patient demographics and presentation similar to cases in literature coupled with a positive Widal test strengthened our suspicion of Salmonellae Typhi as the causative organism.

Salmonellae reach the gallbladder through the bloodstream and have been shown to have a tropism for the vesicular gallbladder wall.^7^ In acute typhoid infections, colonization of the gallbladder is rarely diagnosed but becomes apparent when features of acalculous cholecystitis develop.

The gallbladder acts as a unique replication niche for salmonellae which explains the high concentration of bacteria in infected gallbladders. Salmonella is known to undergo both extracellular replication in bile as well as intracellular replication in the epithelial cells of the gallbladder wall. This causes the sloughing of infected cells, inflammation, and tissue damage.^7^ Intense inflammation of the gallbladder coupled with infection with a virulent organism like Salmonellae in an immunocompromised individual leads to thrombosis of the cystic artery with transmural necrosis and perforation.^8^

The patient had matted inflamed and edematous bowel with free pus in the abdomen which was thought to either be due to a sealed perforation since she had been on antibiotics or due to leakage of the pericholecystic abscess.^5^ Previous reports have shown that complicated acalculous cholecystitis can occur in concordance with bowel complications though this could not be confirmed in this patient.^9^

Indications for cholecystectomy in children with acute acalculous cholecystitis are gallbladder complications like perforation, empyema, and gangrene commonly seen in patients with systemic bacterial infections.^10^ Surgery was the treatment of choice in this patient; however due to difficulties in dissection of Calot's triangle, we opted to do a subtotal cholecystectomy with nonclosure of the cystic duct and subhepatic drainage. Though nonclosure of the cystic duct is associated with bile leak in up to 42% of the patients, we believe it was a safer option for our patient.^11^ Since there was no distal blockage of the bile duct, conservative management was sufficient in treating the bile leak which subsequently resolved.

## 4 CONCLUSION

Increased suspicion of S. Typhi cholecystitis especially in typhoid fever patients by attending physicians will enable the use of available diagnostic tools like ultrasound scan coupled with clinical findings for early diagnosis and better patient management. In cases of difficult Calot's triangle dissection, subtotal cholecystectomy should be considered as an operative option.

## ACKNOWLEDGMENT

Consent statement: Published with written consent of the patient.

## CONFLICT OF INTEREST

None declared.

## AUTHOR CONTRIBUTIONS

AVV and DB: involved in patient management and acquisition of the data. AVV and JW: drafted the manuscript. SR: edited the manuscript and gave approval of final copy of manuscript.

## Data Availability

These data are available in public repositories that issue data sets with DOIs.
